# Development of Zein–PEG400/PVA–Chitosan Bilayer Films for Intelligent Packaging

**DOI:** 10.3390/polym17030387

**Published:** 2025-01-31

**Authors:** Rong Sun, Liangliang Li, Jiangjie Zhou, Yongfeng Zhang, Haiya Sun, Datong Zhang, Qi Wu

**Affiliations:** 1 Department of Chemistry, Zhejiang University, Hangzhou 310058, China; 115069@zust.edu.cn; 2 Key Laboratory of Chemical and Biological Processing Technology for Farm Products of Zhejiang Province, School of Biological and Chemical Engineering, Zhejiang University of Science and Technology, Hangzhou 310023, China; 1220450025@zust.edu.cn (L.L.); yuzulemon@163.com (J.Z.); 15970076156@163.com (Y.Z.); 119012@zust.edu.cn (H.S.); 3 Hangzhou Hydrotech Co., Ltd., Hangzhou 311500, China

**Keywords:** bilayer film, zein, anthocyanin, intelligent packaging

## Abstract

Zein exhibits excellent biodegradability, thermal stability, UV resistance, and water barrier properties, making it a promising candidate for food packaging applications. However, pure zein films suffer from brittleness and poor mechanical strength, which limit their practical use. In this study, a unique bilayer packaging film (ZP/P-C) was developed using a layer-by-layer solution casting technique, where hydrophobic zein was coated onto a polyvinyl alcohol and chitosan composite layer (P-C). Incorporating PEG400 into the zein layer improved the interfacial compatibility of the bilayer film, increasing its uniformity and toughness. The resulting bilayer films demonstrated enhanced mechanical properties, flexibility, and water vapor barrier performance. Specifically, the ZP_7.5_/P-C bilayer film showed an elongation at break of 68.74% and a modulus of elasticity of 187.19 MPa. It had a water vapor permeability of 6.60 × 10^−11^ g·m·m^−2^·s^−1^·Pa^−1^ and provided near-complete UV protection within the 200–350 nm range. Furthermore, an intelligent detection bilayer film was created by integrating anthocyanin extract into the zein layer. Adding anthocyanin improved the film’s antioxidant properties and allowed it to respond colorimetrically to total volatile basic nitrogen. The bilayer film ZP_BA1.0_/P-C displayed an excellent antioxidant activity (45.8%) and remarkable color change (ΔE = 20.2) in response to ammonia, effectively indicating shrimp spoilage in 48 h (ΔE > 10). This investigation spotlights the potential of zein-based bilayer films in active and intelligent food packaging, offering innovative strategies to improve food safety and extend the shelf life of perishable goods.

## 1. Introduction

Food packaging materials are essential for maintaining food quality and ensuring safety throughout the supply chain [1]. Conventional synthetic plastics, such as low-density polyethylene, polyvinylidene chloride, and polyvinyl chloride, have found widespread application in the packaging industry due to their durability, low cost, and ease of production. However, these materials are non-biodegradable, contributing significantly to environmental pollution and raising global sustainability concerns [2]. To address these issues, there is an increasing trend toward developing environmentally friendly, biodegradable polymer films as sustainable alternatives [3,4]. Of these, natural biodegradable polymers, including gelatin, starch, sodium alginate, chitosan, and zein, have attracted significant interest for their non-toxic nature, biodegradability, and excellent film-forming properties [5,6].

With excellent film-forming ability, natural biodegradability, and stability under high temperature and humidity conditions, zein has been established as a promising candidate for the production of food-grade and biodegradable packaging material [7]. However, pure zein films exhibit certain limitations, including high opacity, surface hydrophobicity, brittleness, low mechanical strength, and inadequate antibacterial and antioxidant properties, which are critical for modern packaging requirements [8].

Recent studies have focused on overcoming these limitations through blending and modification strategies. Blending zein with polysaccharides, lipids, or other proteins has been shown to improve its mechanical strength, flexibility, and overall functionality [9]. Additionally, modifying the preparation process by adjusting pH, ionic strength, temperature, or incorporating plasticizers has further enhanced film performance [10,11]. Among the materials used to complement zein, chitosan and polyvinyl alcohol (PVA) stand out for their unique and synergistic properties. Chitosan (CS) is a highly versatile material known for its excellent biocompatibility, biodegradability, and antibacterial properties, enabling its applications in wound dressings, drug delivery systems, and environmentally friendly packaging [12,13,14]. The incorporation of chitosan into zein film can improve their mechanical strength, antimicrobial efficacy, and overall stability [15,16]. Meanwhile, PVA is widely recognized for its remarkable film-forming ability, transparency, flexibility, and mechanical durability. Additionally, it demonstrates excellent mechanical strength, outstanding gas barrier properties, and complete biodegradability, positioning it as an eco-friendly and sustainable material for packaging and various applications [17,18]. Blending PVA and chitosan with zein can significantly improve the mechanical strength, antimicrobial activity, and permeability of composite films [19,20]. However, the simple blending of PVA and CS into zein films often involves challenges, such as poor compatibility, low transparency, and difficulties in achieving uniform film performance [21]. In our study, we also found that PVA and CS cannot dissolve uniformly with zein in the same solvent, and this incompatibility makes it extremely difficult to form a uniform and consistent single-layer film.

To address these challenges, bilayer films with complementary characteristics could be designed to enhance polymer film performance, better satisfying the requirements of food packaging [22]. Bilayer film technology involves creating a bilayer structured membrane by combining two materials with different characteristics or functions through composite processes. This approach effectively integrates the properties of different layers, reducing compatibility issues and thereby enhancing overall performance [23,24,25]. Li et al. designed a bilayer intelligent packaging film Kgm/ZTOEO-Car/Al by using a two-step casting strategy. This bilayer film exhibited excellent mechanical strength, thermal stability, light-blocking properties, and moisture and oxygen permeability. Additionally, it features sustained-release antibacterial and antioxidant activity [26].

Therefore, we prepared a Zein–PEG400/PVA–CS bilayer film (ZP/P-C) using a layer-by-layer solution-casting approach. The bilayer design not only resolved the incompatibility issue but also enhanced the overall mechanical and barrier properties of the composite film. This separation of layers enabled us to optimize the properties of each individual material—such as flexibility, strength, and barrier performance—ensuring a higher quality film. The bilayer configuration exploited the complementary properties of the individual layers: the zein layer contributed water resistance and UV barrier properties [27], while the P-C layer enhanced mechanical strength, flexibility, and antibacterial performance. Furthermore, eugenol was incorporated to reduce the inherent odor of the zein layer while enhancing its antimicrobial and oxidative stability. Building on this foundation, a smart packaging system was developed by incorporating anthocyanins into the ZP layer. Anthocyanins are pH-sensitive natural pigments with strong antioxidant activity, enabling the packaging to visually indicate food quality by exhibiting color changes in response to total volatile basic nitrogen [28]. This bilayer film thus serves as an intelligent indicator of seafood freshness, providing a real-time chromatic response to spoilage.

## 2. Materials and Methods

### 2.1. Materials

Polyethylene Glycol (PEG400), chitosan (CS, Mw. 50 kDa, deacetylation degree ≥95%), eugenol (Eug, purity 99%), zein, and polyvinyl alcohol (PVA-1788) were purchased from Macklin Biochemical Technology Co., Ltd. (Shanghai, China); blueberry anthocyanin (BA, purity 5~25%) was purchased from Yuanye Biotechnology Co., Ltd. (Shanghai, China). 1,1-diphenyl-2-picrylhydrazyl (DPPH) was purchased from Aladdin Biochemical Technology Co., Ltd. (Shanghai, China).

### 2.2. Preparation of ZP/P-C Bilayer Films

We obtained 4% (*w*/*v*) PVA solution by way of stirring continuously in deionized water for 2 h at 95 °C. To prepare a 3% (*w*/*v*) chitosan solution, the chitosan was dissolved in a 1% (*v*/*v*) aqueous solution of acetic acid. A 20% (*w*/*v*) zein solution was made by mixing zein with an 80% (*v*/*v*) ethanol/water solution. The zein solution and P-C solution were prepared according to the formulas in Table 1. Eug was the odor regulator. Single-layer Zein and P-C films were, respectively, prepared by casting 4 g ZP solution and 8 g P-C solution onto 10 × 10 cm dishes, removing solvent for 12 h at 50 °C. The ZP/P-C bilayer films were fabricated through a two-step process. Initially, a P-C single layer was cast and allowed to solidify. Then, 4 g of ZP solution was uniformly poured onto the surface of the P-C layer. Each layer was desolventized at 50 °C for 12 h.

### 2.3. Preparation of ZP_BA_/P-C Bilayer Indicator Films

To obtain the intelligent indication function of the film, BA were added in ZP_7.5_ solution to prepare single-layer ZP_BA_ and bilayer film ZP_BA_/P-C. The formulas of the bilayer indicator films were shown in Table 2. The films were prepared following the same procedure described in Section 2.2.

### 2.4. Characterizations

#### 2.4.1. Fourier Transform Infrared Spectroscopy Analysis

The chemical structure of single-layer and bilayer films were measured using Fourier Transform Infrared Spectrometer (FTIR, VERTEX 70, Bruke, Karlsruhe, Germany) at room temperature, with potassium bromide as the background. Absorption spectra were recorded in the range of 4000–400 cm^−1^.

#### 2.4.2. X-Ray Diffractometer Analysis

The crystallinity of the films was investigated using an X-ray diffractometer (XRD, Ultima IV, Rigaku, Tokyo, Japan). Samples (15 × 15 mm) were tested with Cu Kα radiation (λ = 0.154 nm), at 40 mA current, 40 kV voltage, a scanning speed of 5°/min, and a scanning range of 5–45 °C.

#### 2.4.3. Scanning Electron Microscopy Analysis

Surface and cross-section morphology of the films were observed using a scanning electron microscope (SEM, SU 1510, Hitachi, Tokyo, Japan) at 15 kV. Samples were coated in gold for 120 s.

#### 2.4.4. Mechanical Properties Analysis

The films were cut into 70 × 10 mm pieces, and their mechanical properties were measured using a Universal Testing Machine (CMT 4104, Sansi, Shanghai, China) with a crosshead speed of 5 mm/min. The tensile strength at break was TS = F_max_/A and the elongation at break was EB = (L/L_0_) × 100%, where F is the breaking load, A is the initial cross-sectional area of the sample (thickness × width), L_0_ is the original length of the sample, and L is the increase in length at breaking point.

#### 2.4.5. Water Vapor Transmittance Test

All of the films were cut into 60 × 60 mm pieces. The sample was fixed in a moisture-permeable cup containing anhydrous calcium chloride and placed in an oven with constant temperature and humidity (90%). The weight change in the moisture-permeable cup was measured over 48 h [29], and the water vapor transmission rate (WVP) was calculated according to the equation below:

WVP = (Δm × d)/(A × t × ΔP)(1)

where Δm (kg) = the weight increase of the test cup, d (m) = the average film thickness, A (m^2^) = the exposed film surface area, t (s) = the equilibration time, and ΔP (Pa) = the water vapor pressure difference across the film.

#### 2.4.6. Wetting Angle Analysis

The wetting angle of water on the film surface was measured using a Drop Shape Analyzer (DSA 30E, KRUSS, Hamburg, Germany). After 5 μL of ultrapure water droplets were placed on the film, the image was captured, and the contact angle recorded. The average value was taken from three measurements.

#### 2.4.7. Light Transmittance and Haze Measurement

The light transmittance and haze of the films were measured using a light transmittance haze tester (SGW 820, Shanghai Instruments, Shanghai, China), while UV transmittance was determined with a UV2600 Spectrophotometer (Shimadzu, Kyoto, Japan).

#### 2.4.8. Antioxidant Analysis

The antioxidant activity was determined by DPPH analysis [29]. A total of 60 mg of the film was placed in 3 mL of methanolic DPPH solution (0.1 mmol/L) and incubated in the dark at room temperature for 30 min. Subsequently, the absorbance at 516 nm was measured using a UV 2600 Spectrophotometer (Shimadzu, Kyoto, Japan). The DPPH radical scavenging activity was calculated as follows:

Antioxidant activity (%) = (A_0_ − A_1_)/A_0_ × 100% (2)

where A_0_ represents the absorbance of the control solution, while A_1_ is the absorbance of the test film. Three replicates were measured for each film.

#### 2.4.9. Ammonia-Responsive Color Indication Performance

The films (25 × 25 mm) were individually attached to glass dishes. Place the dishes together with a beaker containing 400 mL of 10% ammonia solution in a sealed container. The color change in the films was observed by photographing after treatment for 0.5 h, 1 h, 2 h, 4 h, and quantitatively analyzed using a Chroma Meter CR-400 (Konica Minolta, Tokyo, Japan) [30]. The color difference value (ΔE) was calculated according to equation:

(3) $$\Delta E=\sqrt{{(L-L_{0})}^{2}+{(a-a_{0})}^{2}+{(b-b_{0})}^{2}}$$

where L, a, and b represent the brightness, red–green, and yellow–blue chromaticity of the film at different times, respectively, and L_0_, a_0_, and b_0_ denote their initial values.

#### 2.4.10. Practical Monitoring of the Spoilage of Shrimp Using the ZP_BA_/P-C

Approximately 25 g of fresh shrimp were placed on a clean Petri dish and covered with Z_BA_/P-C film, and control samples were applied separately. The dish was then sealed with tape and stored at 25 °C for 72 h. Color changes in the Z_BA_/P-C film were monitored as an indicator of shrimp freshness, and the calculation of ΔE followed the method described in Section 2.4.9.

#### 2.4.11. Total Volatile Basic Nitrogen

To evaluate the freshness of shrimp, an analysis of total volatile basic nitrogen (TVBN) content was performed [31]. The procedure involved homogenizing 10 g of shrimp in pure water for a duration of 30 min. Then, 5 mL of the filtrate was placed in the reaction chamber of a distillation apparatus, and 5 mL of a 1% magnesium oxide (MgO) solution was introduced. The distillate was collected in a 2% boric acid solution. After the reaction, the solution was titrated with 0.01 mol/L hydrochloric acid to the endpoint. The TVBN content was then determined by calculating the volume of hydrochloric acid used, expressed in mg/100 g, following the equation:

TVBN (mg/100 g) = (V × N × 14.01)/g × 100(4)

where V = the volume of HCl used in titration (mL), N = the equivalent concentration of HCl, m = the sample weight, and 14.01 = molecular weight of nitrogen.

## 3. Results and Discussions

### 3.1. Structure Analysis

**Figure 1:**
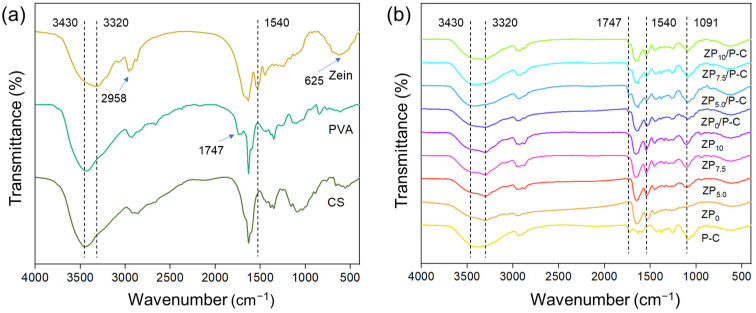
FTIR spectra of zein, PVA, CS (**a**) and different films (**b**).

Figure 1 illustrated the FTIR spectra of all the films. The spectrum of zein exhibited peaks at 3320 cm^−1^, 2958 cm^−1^ (-CH_3_ stretching), 1650 cm^−1^ (C=O stretching), 1540 cm^−1^ (Benzene skeleton), 625 cm^−1^ [32]. The single-layer P-C film demonstrated a composite spectral profile characteristic of PVA and CS, with distinct peaks observed at 1747 cm^−1^, attributable to the PVA signature peak, and 1091 cm^−1^, corresponding to the CS signature peak [33]. Meanwhile, the single-layer films ZP_0_, ZP_5.0_, ZP_7.5_ and ZP_10_ exhibited analogous peak positions; however, with an increasing concentration of PEG400, there was a progressive enhancement in peak intensity of 2920–2750 cm^−1^, associated with the C-H stretching vibrations in the CH_2_ groups of PEG400, and at 1100 cm^−1^, corresponding to the C-O-C stretching vibrations [34]. Furthermore, the ZP/P-C bilayer films not only mirrored the characteristic peaks of the single-layer ZP films but also exhibited augmented peak intensity at 3430 cm^−1^. Additionally, there was a partial superposition of peaks at 1100 and 1091 cm^−1^ in the bilayer film, which result in heightened peak intensity at these specific wavenumbers.

### 3.2. Morphological Characterization

**Figure 2:**
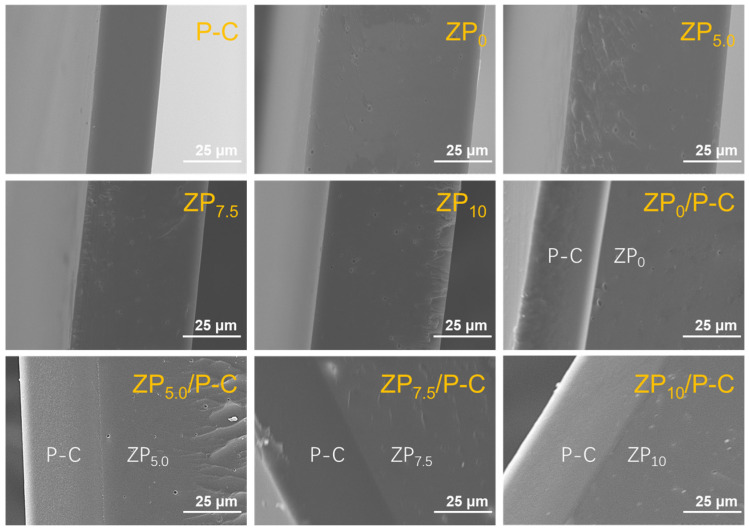
Cross-section SEM images of the single-layer and bilayer films.

The surface and cross-sectional microstructures of the films were shown in Figure S1 and Figure 2. The surfaces of both single-layer films and bilayer films were homogeneous, smooth and dense, indicating a well compatibility between P-C and zein, as well as PVA and CS. Meanwhile, it was observed that the P-C film exhibited a dense cross-section without porosity [35]. However, the bilayer films revealed a distinct two-phase interface, which was attributed to the incompatibility stemming from the divergent physical properties of P-C and zein (Figure 2) [36]. The ZP_0_/P-C film displayed pronounced phase separation, characterized by the presence of micro-fissures at the interlayer junction. With an increased content of PEG400, these micro-fissures were observed to gradually disappear, and the interface became less distinct. The ZP_7.5_/P-C and ZP_10_/P-C films showcased a tightly integrated micro-interfacial structure, indicating improved interfacial compatibility and the establishment of a more robust bilayer film. This improvement was attributed to the formation of strong hydrogen bonds between the ether and hydroxyl groups of PEG and the hydroxyl or amine group of zein, PVA, and chitosan. These interactions improve the interlayer adhesion and lead to a more uniform film structure. Additionally, within the bilayer film construct, the P-C layer preserved its compact structure, while the zein layer presented a notably rough cross-sectional appearance, accompanied by a sparse distribution of pores, which potentially enhanced the film’s gas permeability [24].

### 3.3. XRD Analysis

**Figure 3:**
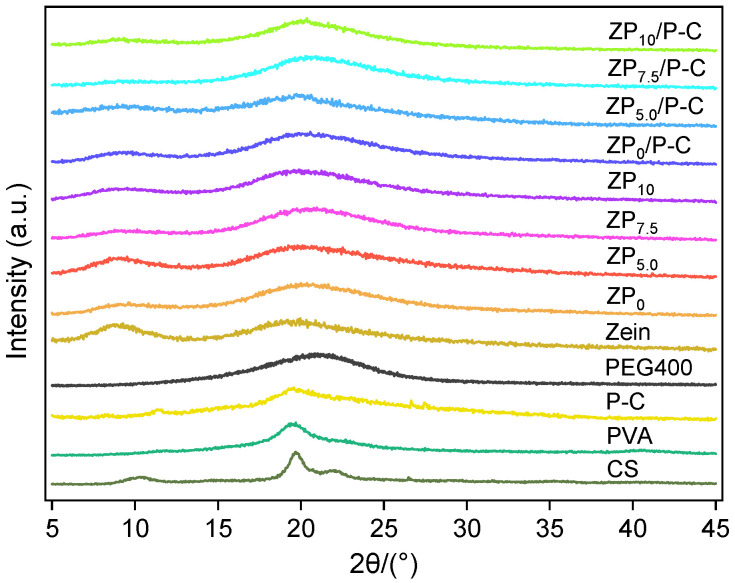
XRD spectra of different single-layer and bilayer films.

The XRD patterns of Zein, CS, PVA and all single-layer films and bilayer films were shown in Figure 3. PVA exhibited crystalline peak at 2θ = 19.6° ascribe to the (101) reflection [36]. Crystalline peaks of CS were observed at 2θ = 10.2° and 19.7° [33], and the similar moderate humps of zein and single-layer ZP films exhibited the amorphous property. The intensity of the peaks was significantly lower in the P-C film than in the PVA, which was attributed to the weakening of intermolecular interactions due to the mixing CS, resulting in decreased crystallinity. It was worth noting that with the addition of PEG400, the 2θ of the broad peaks of single-layer ZP films and bilayer films were slightly increased, possibly attributed to the overlap of the PEG400 peak. Furthermore, the diffraction peaks of the bilayer films exhibited a resemblance to those of the ZP single-layer films, due to the overlapping peaks of P-C and ZP layer, coupled with the relatively lower content of P-C.

### 3.4. Mechanical Properties

The elongation at break (EB), tensile strength (TS), and modulus of elasticity (ME) reflect the flexibility and mechanical strength of the food packaging materials. The mechanical properties of the films were presented in Table 3. ZP_0_ film exhibited higher TS (30.26 MPa) and ME (1674.88 MPa) than P-C film (24.58 MPa and 867.29 MPa, respectively), but it had notably lower EB (2.45%) than P-C film (45.12%). As the content of PEG400 increased, the single-layer zein films exhibited an increased EB, accompanied by a gradual decrease in TS and ME. The EB of ZP_7.5_ was 47.99%, with TS and ME being 1.67 MPa and 78.98 MPa, respectively. PEG400 could effectively disperse within the zein molecular chains by forming hydrogen bonds between its hydroxyl and ether groups and the amine or hydroxyl groups on zein. This interaction reduced intermolecular forces, thereby increasing molecular mobility and improving the flexibility of the film [37]. However, the mechanical resistance was also significantly reduced by adding PEG400. The bilayer film formed by the combination of P-C and ZP layers exhibited superior mechanical strength compared to the single-layer zein film, while also offering enhanced flexibility than the P-C film, thus synergistically enhancing its mechanical properties [38]. Therein, the EB of ZP_7.5_/P-C reached 68.74%, with TS and ME values being 8.98 MPa and 187.19 MPa, respectively. These improved mechanical properties rendered them well-suited for food packaging applications.

### 3.5. Wettability Analysis

**Figure 4:**
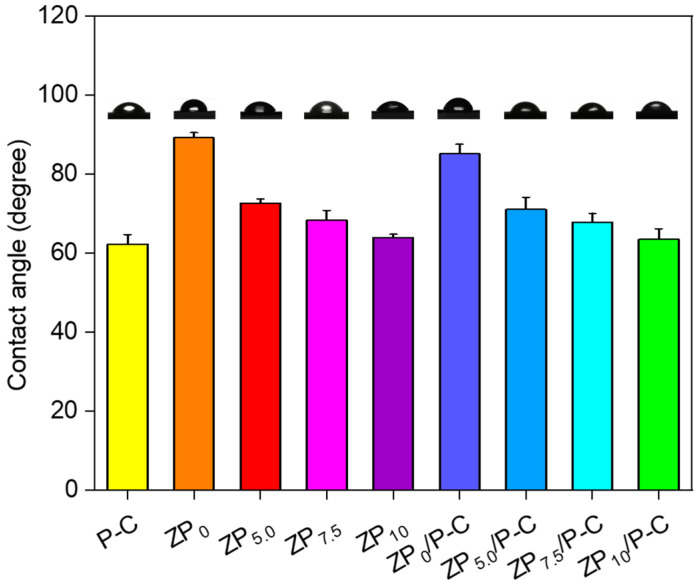
Contact angle of the different films.

The hydrophobicity of the films was evaluated using the water contact angle (WCA), with the results displayed in Figure S2 and Figure 4. The WCA of PVA and CS were 56.5° and 76.6°, respectively (Figure S2), while the WCA of the P-C film was 62.3°. This indicated that the P-C film exhibited intermediate hydrophilicity between PVA and CS, reflecting the combined effect of the two materials within the composite structure [39]. When compounded with ZP_0_, the water contact angle of ZP_0_/P-C bilayer increased significantly from 62.3° to 85.2, revealing the higher hydrophobicity of ZP_0_ [26]. As the PEG400 content increased from 0 to 10%, the WCA of the bilayer films gradually decreased from 85.2° to 63.5°. This could be due to the strengthened hydrogen bonding between PEG400 and zein, as well as the disruption of the compact arrangement of zein molecules, resulting in a reduction in surface free energy and the enhancement of the hydrophilicity of the films. This phenomenon was also reflected in the moisture content (MC) and water solubility (WS) of the films (Figure S3). The P-C film exhibited the highest MC and WS, while ZP_0_ showed the lowest values. Moreover, both the MC and WS of ZP films gradually enhanced with the increase in PEG400 content. Furthermore, bilayer films demonstrated higher WS than single-layer ZP films, which could be ascribed to the higher hydrophilicity of P-C layer.

### 3.6. Water Vapor Transmittance Analysis

**Figure 5:**
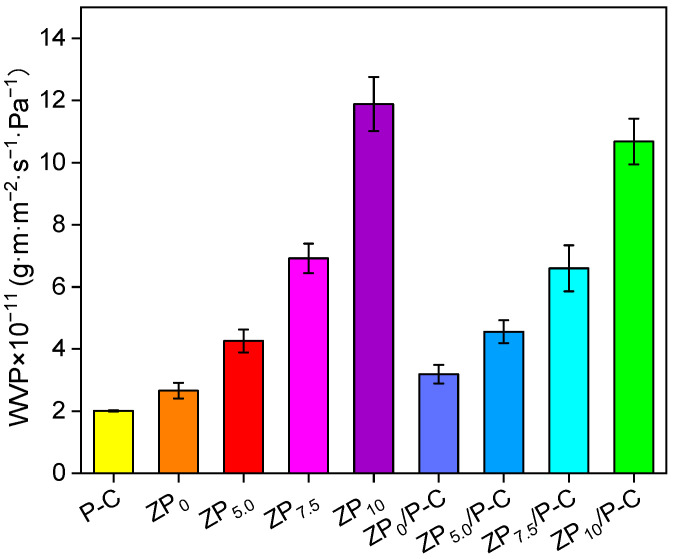
Water vapor permeability of the films.

WVP is a crucial indicator of film’s resilience against the elevated humidity typically encountered on the surfaces of many foods and within their ambient surroundings, thereby providing a crucial benchmark for assessing the film’s moisture barrier capabilities [39]. The WVP of pristine PVA and CS film was 3.39 and 1.91 × 10^−11^ g·m·m^−2^·s^−1^·Pa^−1^, respectively (Figure S4), attributed to the higher hydrophilicity of PVA compared to CS. The WVP of the P-C film was 2.01 × 10^−11^ g·m·m^−2^·s^−1^·Pa^−1^, which lied between those of PVA and CS, indicating that the P-C composite film exhibited a balanced performance in water vapor permeability. As shown in Figure 5, the P-C film exhibited the lowest WVP, which was associated with film thickness, where an increase in thickness corresponds to a higher WVP [33]. Significantly, ZP_0_ demonstrated a higher WVP than P-C film, while WCA data revealed that ZP_0_ possesses a higher hydrophobicity than P-C, which could be attributed to the presence of micropores within the zein film that facilitated the transport of water vapor [26]. Furthermore, the WVP increased progressively with the addition of PEG400, due to its hydrophilic property, which enhances the absorption and transport of water molecules. In comparison to single-layer ZP films, the bilayer films exhibited reduced WVP values, highlighting that the incorporation of P-C contributed to enhanced water vapor barrier properties [40].

### 3.7. Light Transmittance

**Figure 6:**
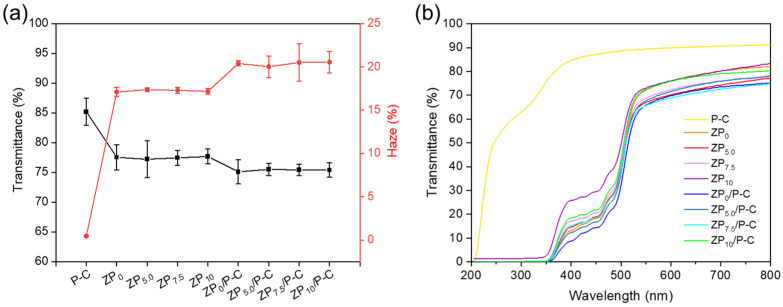
(**a**) Light transmittance and haze of the films; (**b**) UV-vis spectra of the films.

Transmittance and haze are very important indicators for evaluating the transparency performance of films, which are used in evaluating the appearance potential for practical packaging applications. As depicted in Figure 6a, the P-C film demonstrated good light transmittance (85%) and low haze (0.5%), whereas single-layer zein films indicated lower transmittance (approximately 77%) and higher haze (nearly 17%), and the films showed analogous light transmission as the PEG400 content increased, potentially due to the incomplete dissolution of minor impurities within the zein. Compared to single-layer films, the transmittance of bilayer composite films slightly decreased to 75%, while the haze increased to 20%, which illustrated that the composite of P-C exerted minimal influence on the transparency of bilayer films. Additionally, the capacity to block ultraviolet-visible (UV-vis) light plays a vital role in preventing food oxidation induced by UV-vis light to extend food storage [41,42]. Figure 6b presented the UV-vis absorption spectra for all the films. It could be observed that the P-C film exhibited a weaker UV-vis light barrier, while the zein films demonstrated a significant UV-vis light resistance, particularly showing excellent UV radiation blocking within the 200–400 nm wavelength range [29]. Compared to single-layer films, bilayer films possessed superior UV resistance, which was attributed to the combined barrier effects of both zein and P-C [27].

### 3.8. Antioxidant Activity

**Figure 7:**
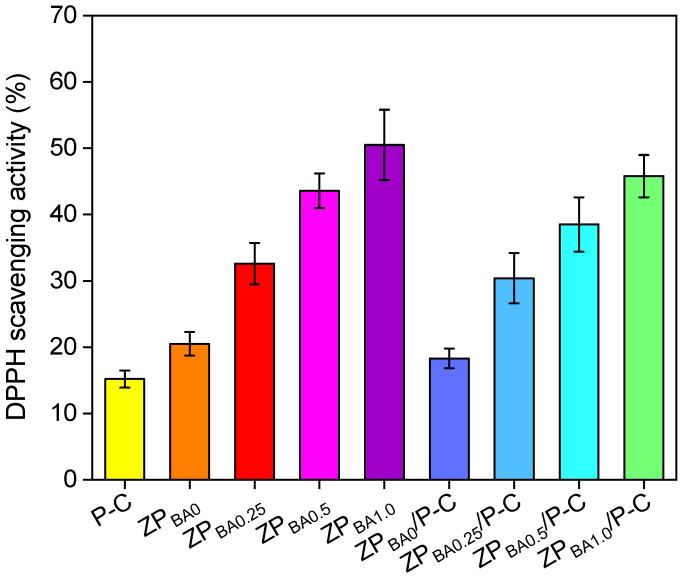
Antioxidant activity of the films.

The aforementioned results indicated that the tensile properties of zein films were significantly improved as the PEG400 content increased, while the hydrophilicity and water vapor permeability also showed a notable rise. Upon a comprehensive evaluation of the mechanical and barrier properties of the films, the ZP_7.5_/P-C bilayer film exhibited the most appropriate performance. To obtain a functional bilayer film with spoilage indication capability, we incorporated blueberry anthocyanins into the ZP_7.5_/P-C film. This anthocyanin exhibits pH-stimuli color-changing behavior, which can indicate the spoilage of high-protein foods. In addition, blueberry anthocyanins also possess excellent antioxidant activity. The evaluation of antioxidative capacity is essential for understanding a material’s ability to neutralize and eliminate free radicals. Herein, we determined the antioxidant activity of the films by using the DPPH radical scavenging method, and the results were illustrated in Figure 7. The antioxidant activity of P-C and ZP_BA0_ films was relatively low. However, as the BA content increased, the DPPH radical scavenging activity showed a gradual enhancement in both single-layer zein films and bilayer films [43,44]. The DPPH scavenging activity of the ZP_BA0_ and ZP_BA0_/P-C film were 20.5% and 18.3%, respectively, which could be attributed to the eugenol in zein layer. In contrast, the antioxidant activity of the ZP_BA1.0_ and ZP_BA1.0_/P-C films exhibited a significant increase, reaching 50.5% and 45.8%, respectively, which can be ascribed to the antioxidant properties of anthocyanins.

### 3.9. Ammonia-Responsive Color Indication of ZP_BA_/P-C Film

**Figure 8:**
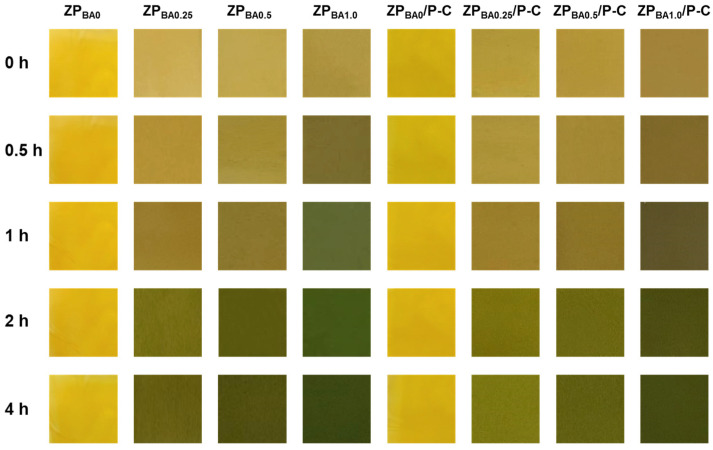
Color change in the films under different exposure time of NH_3_ vapor.

Protein-rich meats and seafood may produce ammonia and various amine-based alkaline nitrogenous compounds, during spoilage caused by enzymatic and bacterial activity [45,46]. Therefore, ammonia (NH_3_) was used as a representative of the volatile basic gases produced by food spoilage to investigate the response of the indicator film [47,48]. Figure 8 showed images of the indicator film exposed to NH_3_ over different periods. As time extends, the indicator film exhibited gradually color changes from yellow to dark green, and the colorimetric change was more obviously with an increase in the content of BA [49]. The ZP_BA1.0_ and ZP_BA1.0_/P-C films demonstrated the most pronounced color changes. These color changes are easily noticeable to the naked eye, suggesting that the films exhibit excellent responsiveness and sensitivity to volatile basic nitrogen compounds. The reaction principle involves the interaction of nitrogenous gases with water in the thin film, resulting in hydrolysis that generates hydroxide ions, which then deprotonate anthocyanins, forming anionic quinonoidal and chalcone structures, leading to the color transition [50].

**Figure 9:**
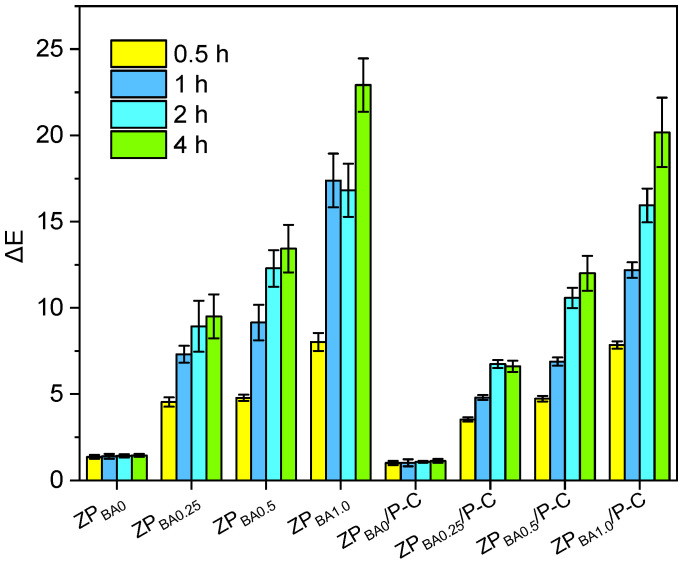
The color difference value of the films.

The ΔE value indicates the color difference from the original color of the film [51]. A color change with ΔE > 3 is readily noticeable to the naked eye [52]. Figure 9 illustrated that the ΔE of the film without BA remained essentially constant, while the ΔE of the film containing BA gradually increased over time. After 1 h, this increase slowed down. Furthermore, the increase in ΔE became more significant with an increase in BA content. After 4 h in a volatile NH_3_ atmosphere, the ΔE value of ZP_BA1.0_ and ZP_BA1.0_/P-C films reached 22.9 and 20.2, respectively. Additionally, the bilayer film exhibited a slightly lower ΔE compared to the single-layer film, potentially attributed to the combined influence of the P-C layer on the film’s permeability to NH_3_ and water vapor.

### 3.10. Practical Monitoring of the Spoilage of Shrimp

**Figure 10:**
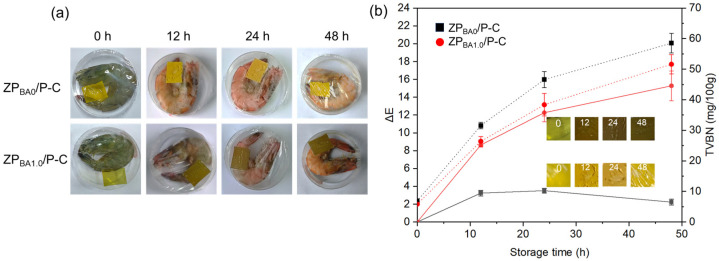
(**a**) Color changes in shrimp with ZP_BA0_/P-C and ZP_BA1.0_/P-C bilayer films at storage intervals of 0 h, 12 h, 24 h, and 48 h; (**b**) The ΔE and TVBN values of ZP_BA0_/P-C and ZP_BA1.0_/P-C films during the storage period for packaged shrimp. The solid line represents ΔE, and the dashed line represents TVBN.

To assess the efficacy of bilayer indicator films in indicating spoilage in high-protein meat products, we monitored the release of TVBN from shrimp over various time intervals and observed corresponding color changes in the films [53,54]. The acceptable TVBN levels for shrimp are categorized as follows: fresh shrimp with TVBN below 12 mg/100 g, slightly spoiled but still edible shrimp with 12–20 mg/100 g, borderline cases fall within 20–30 mg/100 g, while shrimp with TVBN levels exceeding 30 mg/100 g are deemed inedible and spoiled [29]. Figure 10 illustrated the visual color alterations of the films and the associated TVBN values during shrimp storage at 30 °C across different time frames. ZP_BA0_/P-C film devoid of anthocyanins served as control samples. A marked contrast in response to shrimp spoilage was observed between the control films and those containing anthocyanins. As the control film, ZP_BA0_/P-C exhibited a slight color change, with a ΔE of approximately 2.23 at 48 h, primarily attributed to the films’ water absorption. In contrast, the anthocyanin-containing ZP_BA1.0_/P-C film demonstrated a noticeable color change within 12 h of storage, and this change became more pronounced with increased storage time, reaching a ΔE of 15.27 at 48 h. The color change was closely correlated with the concentration of TVBN. At 12 h, the TVBN value for ZP_BA1.0_/P-C reached 26.38 mg/100 g, indicating advanced spoilage. By 24 h, the TVBN had further increased to 38.35 mg/100 g, signifying spoilage. The TVBN rose significantly to 54.60 mg/100 g at 48 h, accompanied by the color of ZP_BA1.0_/P-C film turning dark green. These changes confirmed that the shrimp had spoiled after being stored for 24 h at 30 °C, exceeding the edibility threshold. The ZP_BA0_/P-C group demonstrated a similar increasing trend in TVBN values to that observed in the ZP_BA1.0_/P-C group. These observations confirmed that the shrimp had spoiled beyond the edible threshold within merely 12 h of storage at 30 °C. The escalation in TVBN values is primarily attributed to the accumulation of nitrogenous compounds resulting from microbial proliferation [55]. Collectively, these results emphasize the capability of the prepared films to assess the quality and freshness of packaged shrimp.

## 4. Conclusions

In this work, a ZP/P-C bilayer film with enhanced mechanical and barrier performance was developed through a layer-by-layer solution casting method, intended for intelligent food packaging applications. Incorporating PEG400 with zein increased the plasticity and hydrophilicity of the zein layer while enhancing interlayer compatibility between the zein and P-C layers, resulting in a uniform film structure. Simultaneously, the bilayer films exhibited excellent UV resistance and improved mechanical strength and water vapor barrier performance. Moreover, the inclusion of anthocyanins conferred to the bilayer films remarkable antioxidant properties and the color-changing attribute, corresponding to sensitivity to TVBN (ΔE > 10), which are essential for intelligent packaging that can delay food oxidative degradation and facilitate instant tracking of food freshness. Overall, this study underscores the exceptional performance of bilayer-responsive films, presenting a promising new avenue for biodegradable and intelligent packaging solutions.

## Figures and Tables

**Table 1:** The formulas for single-layer and bilayer films.

Name	Mass Ratio	
	ZP Solution (20% zein/PEG400/Eug)	P-C Solution (4% PVA/3% CS)
ZP_0_	100/0/2	—
ZP_5.0_	100/5.0/2	—
ZP_7.5_	100/7.5/2	—
ZP_10_	100/10/2	—
P-C	—	1/1
ZP_0_/P-C	100/0/2	1/1
ZP_5.0_/P-C	100/5.0/2	1/1
ZP_7.5_/P-C	100/7.5/2	1/1
ZP_10_/P-C	100/10/2	1/1

**Table 2:** The formulas for single-layer and bilayer indicator films.

Name	Mass Ratio	
	ZP Solution (20% zein/Eug/PEG400/BA)	P-C Solution (4% PVA/3% CS)
ZP_BA0.25_	100/7.5/2/0.25	—
ZP_BA0.5_	100/7.5/2/0.5	—
ZP_BA1.0_	100/7.5/2/1.0	—
ZP_BA0.25_/P-C	100/7.5/2/0.25	1/1
ZP_BA0.5_/P-C	100/7.5/2/0.5	1/1
ZP_BA1.0_/P-C	100/7.5/2/1.0	1/1

**Table 3:** Mechanical properties of the films.

Film Samples	Thickness (mm)	Tensile Strength (MPa)	Modulus of Elasticity (MPa)	Elongation at Break (%)
P-C	0.027 ± 0.002 ^d^	24.58 ± 1.23 ^b^	867.29 ± 10.12 ^d^	45.12 ± 2.13 ^c^
ZP_0_	0.070 ± 0.004 ^b^	30.26 ± 2.12 ^a^	1674.88 ± 21.45 ^i^	2.45 ± 0.23 ^a^
ZP_5.0_	0.065 ± 0.003 ^bc^	8.46 ± 0.45 ^e^	452.45 ± 10.12 ^g^	11.08 ± 0.46 ^e^
ZP_7.5_	0.058 ± 0.003 ^c^	1.67 ± 0.12 ^gh^	78.98 ± 6.13 ^e^	47.99 ± 2.16 ^g^
ZP_10_	0.060 ± 0.003 ^c^	0.76 ± 0.08 ^h^	25.60 ± 2.15 ^a^	150.81 ± 6.25 ^h^
ZP_0_/P-C	0.102 ± 0.004 ^a^	21.51 ± 2.13 ^c^	1052.81 ± 15.16 ^hi^	6.46 ± 0.51 ^b^
ZP_5.0_/P-C	0.105 ± 0.003 ^a^	12.75 ± 1.01 ^d^	536.96 ± 11.28 ^f^	29.75 ± 2.15 ^d^
ZP_7.5_/P-C	0.103 ± 0.002 ^a^	8.98 ± 0.24 ^f^	187.19 ± 6.53 ^c^	68.74 ± 2.56 ^f^
ZP_10_/P-C	0.102 ± 0.004 ^a^	3.12 ± 0.21 ^fg^	24.53 ± 1.47 ^b^	106.86 ± 4.89 ^g^

Data were expressed as means ± standard deviations. Different lowercase letters within the same column denoted statistically significant differences (*p* < 0.05).

## Data Availability

The datasets used and/or analyzed during this study are available from the corresponding author upon reasonable request.

## Supplementary Materials

The following supporting information can be downloaded at: https://www.mdpi.com/article/10.3390/polym17030387/s1: Figure S1: Surface SEM images of the different films; Figure S2: Contact angle of PVA and CS films; Figure S3: Moisture content and solubility of the different films; Figure S4: Water vapor permeability of PVA and CS films.

## References

[1] Satchanska G., Davidova S., Petrov P.D. (2024). Natural and Synthetic Polymers for Biomedical and Environmental Applications. Polymers.

[2] Cheng J., Gao R., Zhu Y., Lin Q. (2024). Applications of Biodegradable Materials in Food Packaging: A Review. Alex. Eng. J..

[3] Hamdani S.S., Li Z., Rolland E., Mohiuddin M., Rabnawaz M. (2023). Barrier and Mechanical Properties of Biodegradable Paper Bilayer-Coated with Plasticized Starch and Zein. J. Appl. Polym. Sci..

[4] Kimna C., Tamburaci S., Tihminlioglu F. (2019). Novel Zein-Based Multilayer Wound Dressing Membranes with Controlled Release of Gentamicin. J. Biomed. Mater. Res. Part B Appl. Biomater..

[5] Asgher M., Qamar S.A., Bilal M., Iqbal H.M.N. (2020). Bio-Based Active Food Packaging Materials: Sustainable Alternative to Conventional Petrochemical-Based Packaging Materials. Food Res. Int..

[6] Atta O.M., Manan S., Shahzad A., Ul-Islam M., Ullah M.W., Yang G. (2022). Biobased Materials for Active Food Packaging: A Review. Food Hydrocoll..

[7] Lan X., Zhang X., Wang L., Wang H., Hu Z., Ju X., Yuan Y. (2023). A Review of Food Preservation Based on Zein: The Perspective from Application Types of Coating and Film. Food Chem..

[8] Vahedikia N., Garavand F., Tajeddin B., Cacciotti I., Jafari S.M., Omidi T., Zahedi Z. (2019). Biodegradable Zein Film Composites Reinforced with Chitosan Nanoparticles and Cinnamon Essential Oil: Physical, Mechanical, Structural and Antimicrobial Attributes. Colloids Surf. B Biointerfaces.

[9] Bhaskar R., Zo S.M., Narayanan K.B., Purohit S.D., Gupta M.K., Han S.S. (2023). Recent Development of Protein-Based Biopolymers in Food Packaging Applications: A Review. Polym. Test..

[10] Jaski A.C., Schmitz F., Horta R.P., Cadorin L., da Silva B.J.G., Andreaus J., Paes M.C.D., Riegel-Vidotti I.C., Zimmermann L.M. (2022). Zein—A Plant-Based Material of Growing Importance: New Perspectives for Innovative Uses. Ind. Crops Prod..

[11] Wu H., Wang J., Li T., Lei Y., Peng L., Chang J., Li S., Yuan X., Zhou M., Zhang Z. (2023). Effects of Cinnamon Essential Oil-Loaded Pickering Emulsion on the Structure, Properties and Application of Chayote Tuber Starch-Based Composite Films. Int. J. Biol. Macromol..

[12] Chen Y., Liu Y., Dong Q., Xu C., Deng S., Kang Y., Fan M., Li L. (2023). Application of Functionalized Chitosan in Food: A Review. Int. J. Biol. Macromol..

[13] Aranaz I., Alcántara A.R., Civera M.C., Arias C., Elorza B., Heras Caballero A., Acosta N. (2021). Chitosan: An Overview of Its Properties and Applications. Polymers.

[14] Thambiliyagodage C., Jayanetti M., Mendis A., Ekanayake G., Liyanaarachchi H., Vigneswaran S. (2023). Recent Advances in Chitosan-Based Applications—A Review. Materials.

[15] Wang X., Sun Y., Liu Z., Huang X., Yi F., Hou F., Zhang F. (2021). Preparation and Characterization of Chitosan/Zein Film Loaded with Lemon Essential Oil: Effects on Postharvest Quality of Mushroom (*Agaricus bisporus*). Int. J. Biol. Macromol..

[16] Geng C., Liu X., Ma J., Ban H., Bian H., Huang G. (2023). High Strength, Controlled Release of Curcumin-Loaded ZIF-8/Chitosan/Zein Film with Excellence Gas Barrier and Antibacterial Activity for Litchi Preservation. Carbohydr. Polym..

[17] Verma C., Quraishi M.A. (2022). Polyvinyl Alcohol (PVA) as a Biodegradable Polymeric Anticorrosive Material: A Review on Present Advancements and Future Directions. Corros. Eng. Sci. Technol..

[18] Suleiman G.S.A., Zeng X., Chakma R., Wakai I.Y., Feng Y. (2024). Recent Advances and Challenges in Thermal Stability of PVA-Based Film: A Review. Polym. Adv. Technol..

[19] Ma N., Li K., Xu B., Tian H., Ma S., Li J., Ouyang Y., Liu Q., Liu D., Kumar R. (2024). Zein/Polyvinyl Alcohol-Based Electrospun Nanofibrous Films Reinforced with Nano-Hydroxyapatite for Efficient Cu(II) Adsorption. J. Appl. Polym. Sci..

[20] Liu F.S., Zhang X.W., Xiao X.L., Duan Q.F., Bai H., Cao Y.F., Zhang Y., Alee M., Yu L. (2023). Improved Hydrophobicity, Antibacterial and Mechanical Properties of Polyvinyl Alcohol/Quaternary Chitosan Composite Films for Antibacterial Packaging. Carbohydr. Polym..

[21] Bueno J.N.N., Corradini E., de Souza P.R., de S. Marques V., Radovanovic E., Muniz E.C. (2021). Films Based on Mixtures of Zein, Chitosan, and PVA: Development with Perspectives for Food Packaging Application. Polym. Test..

[22] Chen C., Du Y., Zuo G., Chen F., Liu K., Zhang L. (2020). Effect of Storage Condition on the Physico-Chemical Properties of Corn–Wheat Starch/Zein Edible Bilayer Films. R. Soc. Open Sci..

[23] Easdani M., Ahammed S., Saqib M.N., Liu F., Zhong F. (2024). Engineering Biodegradable Controlled Gelatin-Zein Bilayer Film with Improved Mechanical Strength and Flexibility. Food Hydrocoll..

[24] Zhang L., Chen D., Yu D., Regenstein J.M., Jiang Q., Dong J., Chen W., Xia W. (2022). Modulating Physicochemical, Antimicrobial and Release Properties of Chitosan/Zein Bilayer Films with Curcumin/Nisin-Loaded Pectin Nanoparticles. Food Hydrocoll..

[25] Huang Z., Zhai X., Yin L., Shi J., Zou X., Li Z., Huang X., Ma X., Povey M. (2023). A Smart Bilayer Film Containing Zein/Gelatin/Carvacrol and Polyvinyl Alcohol/Chitosan/Anthocyanin for Lateolabrax Japonicus Preservation and Freshness Monitoring. J. Food Meas. Charact..

[26] Li S., Liu L., Yang S., Wang X., Yuan Y., Yue T., Cai R., Wang Z. (2025). Designing Double-Layer Smart Packaging with Sustained-Release Antibacterial and Antioxidant Activities for Efficient Preservation and High-Contrast Monitoring. Food Hydrocoll..

[27] Zhang L., Li K., Yu D., Regenstein J.M., Dong J., Chen W., Xia W. (2022). Chitosan/Zein Bilayer Films with One-Way Water Barrier Characteristic: Physical, Structural and Thermal Properties. Int. J. Biol. Macromol..

[28] Zheng L., Liu L., Yu J., Shao P. (2022). Novel Trends and Applications of Natural PH-Responsive Indicator Film in Food Packaging for Improved Quality Monitoring. Food Control.

[29] Radoor S., Jayakumar A., Karayil J., Kim J.T., Siengchin S. (2024). Nelumbo Nucifera Flower Extract Incorporated Alginate/Polyvinyl Alcohol Films as a Sustainable PH Indicator for Active Food Packaging Applications. Int. J. Biol. Macromol..

[30] Choi Y.H., Kim J.T., Kim M.H., Park W.H. (2024). Biodegradable Poly(3-Hydroxybutyrate-Co-4-Hydroxybutyrate)/Curcumin Composite Film as a Smart Indicator of Food Spoilage. Sens. Actuators B Chem..

[31] Kim H.-J., Roy S., Rhim J.-W. (2022). Gelatin/Agar-Based Color-Indicator Film Integrated with Clitoria Ternatea Flower Anthocyanin and Zinc Oxide Nanoparticles for Monitoring Freshness of Shrimp. Food Hydrocoll..

[32] Hodaei H., Esmaeili Z., Erfani Y., Esnaashari S.S., Geravand M., Adabi M. (2024). Preparation of Biocompatible Zein/Gelatin/Chitosan/PVA Based Nanofibers Loaded with Vitamin E-TPGS via Dual-Opposite Electrospinning Method. Sci. Rep..

[33] Li H., Liu G., Ye K., He W., Wei H., Dang L. (2024). A Novel PH-Sensitive Antibacterial Bilayer Film for Intelligent Packaging. Biomass Convers. Biorefin..

[34] Yu H., Huang X., Zhou L., Wang Y. (2023). Incorporation of Cinnamaldehyde, Carvacrol, and Eugenol into Zein Films for Active Food Packaging: Enhanced Mechanical Properties, Antimicrobial Activity, and Controlled Release. J. Food Sci. Technol..

[35] Yan J., Li M., Wang H., Lian X., Fan Y., Xie Z., Niu B., Li W. (2021). Preparation and Property Studies of Chitosan-PVA Biodegradable Antibacterial Multilayer Films Doped with Cu_2_O and Nano-Chitosan Composites. Food Control.

[36] Liu S., Jiang X., Zhang M., Gao X., Jiang R., Waterhouse G.I.N., Fan H. (2024). Porous Carbon Nitride-Chitosan/Zein Bilayer Functional Films with Efficient Photocatalytic Antibacterial Activity and Tunable Water Barrier Performance for Food Packaging. Food Biosci..

[37] Huo W., Wei D., Zhu W., Li Z., Jiang Y. (2018). High-Elongation Zein Films for Flexible Packaging by Synergistic Plasticization: Preparation, Structure and Properties. J. Cereal Sci..

[38] Avila L.B., Schnorr C., Silva L.F.O., Morais M.M., Moraes C.C., da Rosa G.S., Dotto G.L., Lima É.C., Naushad M. (2023). Trends in Bioactive Multilayer Films: Perspectives in the Use of Polysaccharides, Proteins, and Carbohydrates with Natural Additives for Application in Food Packaging. Foods.

[39] Yao X., Liu J., Hu H., Yun D., Liu J. (2022). Development and Comparison of Different Polysaccharide/PVA-Based Active/Intelligent Packaging Films Containing Red Pitaya Betacyanins. Food Hydrocoll..

[40] Zhang L., Yu D., Xu Y., Jiang Q., Xia W., Yu D. (2023). Changes in Quality and Microbial Diversity of Refrigerated Carp Fillets Treated by Chitosan/Zein Bilayer Film with Curcumin/Nisin-Loaded Pectin Nanoparticles. Food Biosci..

[41] Passaretti M.G., Ninago M.D., Di Anibal C., Pacheco C., Vega D.A., Villar M.A., López O. (2019). V Composite Films with UV Barrier Capacity to Minimize Flavored Waters Degradation. Food Packag. Shelf Life.

[42] Schmitz F., Silva de Albuquerque M.B., Alberton M.D., Riegel-Vidotti I.C., Zimmermann L.M. (2020). Zein Films with ZnO and ZnO:Mg Quantum Dots as Functional Nanofillers: New Nanocomposites for Food Package with UV-Blocker and Antimicrobial Properties. Polym. Test..

[43] Zhang J., Zhang J., Huang X., Arslan M., Shi J., Li Z., Gong Y., Holmes M., Zou X. (2023). Fabrication and Characterization of Polyvinyl Alcohol/Sodium Alginate/Zein/Chitosan Bilayer Film for Dynamic Visualization of Pork Quality. Int. J. Biol. Macromol..

[44] Li S., Wang X., Zhang X., Zhang H., Li S., Zhou J., Fan L. (2023). Interactions between Zein and Anthocyanins at Different PH: Structural Characterization, Binding Mechanism and Stability. Food Res. Int..

[45] Zhang Q., Yu L., Han W., Yang L., Li H., Sun S., Xu Y. (2024). A Self-Calibrating Sensing Platform Based on Amine-Responsive Excitation Wavelength-Dependent Fluorescent Polymers for Real-Time and Visual Detection of Food Freshness. Adv. Funct. Mater..

[46] Dong H., Ling Z., Zhang X., Zhang X., Ramaswamy S., Xu F. (2020). Smart Colorimetric Sensing Films with High Mechanical Strength and Hydrophobic Properties for Visual Monitoring of Shrimp and Pork Freshness. Sens. Actuators B Chem..

[47] Alizadeh-Sani M., Mohammadian E., Rhim J.-W., Jafari S.M. (2020). PH-Sensitive (Halochromic) Smart Packaging Films Based on Natural Food Colorants for the Monitoring of Food Quality and Safety. Trends Food Sci. Technol..

[48] Ai Y., Wang G., Fang F., Zhang F., Liao H. (2022). Development of Real-Time Intelligent Films from Red Pitaya Peel and Its Application in Monitoring the Freshness of Pork. J. Sci. Food Agric..

[49] Li Y., Tang X., Zhu L. (2022). Bilayer pH-Sensitive Colorimetric Indicator Films Based on Zein/Gellan Gum Containing Black Rice (*Oryza sativa* L.) Extracts for Monitoring of Largemouth Bass (*Micropterus salmoides*) Fillets Freshness. Int. J. Biol. Macromol..

[50] Bao Y., Cui H., Tian J., Ding Y., Tian Q., Zhang W., Wang M., Zang Z., Sun X., Li D. (2022). Novel PH Sensitivity and Colorimetry-Enhanced Anthocyanin Indicator Films by Chondroitin Sulfate Co-Pigmentation for Shrimp Freshness Monitoring. Food Control.

[51] Liu J., Huang J., Ying Y., Hu L., Hu Y. (2021). PH-Sensitive and Antibacterial Films Developed by Incorporating Anthocyanins Extracted from Purple Potato or Roselle into Chitosan/Polyvinyl Alcohol/Nano-ZnO Matrix: Comparative Study. Int. J. Biol. Macromol..

[52] Kurek M., Garofulić I.E., Bakić M.T., Ščetar M., Uzelac V.D., Galić K. (2018). Development and Evaluation of a Novel Antioxidant and PH Indicator Film Based on Chitosan and Food Waste Sources of Antioxidants. Food Hydrocoll..

[53] Jia R., Tian W., Bai H., Zhang J., Wang S., Zhang J. (2019). Amine-Responsive Cellulose-Based Ratiometric Fluorescent Materials for Real-Time and Visual Detection of Shrimp and Crab Freshness. Nat. Commun..

[54] Okpala C.O.R., Choo W.S., Dykes G.A. (2014). Quality and Shelf Life Assessment of Pacific White Shrimp (*Litopenaeus vannamei*) Freshly Harvested and Stored on Ice. LWT—Food Sci. Technol..

[55] Kamer D.D.A., Kaynarca G.B., Yücel E., GÜMÜŞ T. (2022). Development of Gelatin/PVA Based Colorimetric Films with a Wide PH Sensing Range Winery Solid by-Product (Vinasse) for Monitor Shrimp Freshness. Int. J. Biol. Macromol..

