# Supplementary material for: Development of Zein–PEG400/PVA–Chitosan Bilayer Films for Intelligent Packaging
Source: Polymers (Basel). 2025 Jan 31;17(3):387. doi: 10.3390/polym17030387 (PMC11820536; doi:10.3390/polym17030387)
Supplement: Supplementary file 1 [file polymers-17-00387-s001.zip › polymers-3432610-supplementary.pdf]

Supplementary information

Development of Zein-PEG400 / PVA-Chitosan Bilayer Films for Intelligent Packaging

Moisture content (MC), water solubility (WS)

The film samples (2 × 2 cm) were initially weighed (W_0) and then dried under vacuum at 70°C for 12 hours, after which their weight was recorded as W_1. The dried films were subsequently immersed in 50 mL of distilled water at 25°C for 10 hours. After soaking, the films were weighed following the removal of surface water, and then vacuum-dried again at 70°C for 12 hours until a constant weight was achieved (W_2). The MC and WS of the films were calculated using the following equations:

$$MC = (W_0 - W_1) / W_0 \times 100\% \quad (1)$$

$$WS = (W_1 - W_2) / W_1 \times 100\% \quad (2)$$

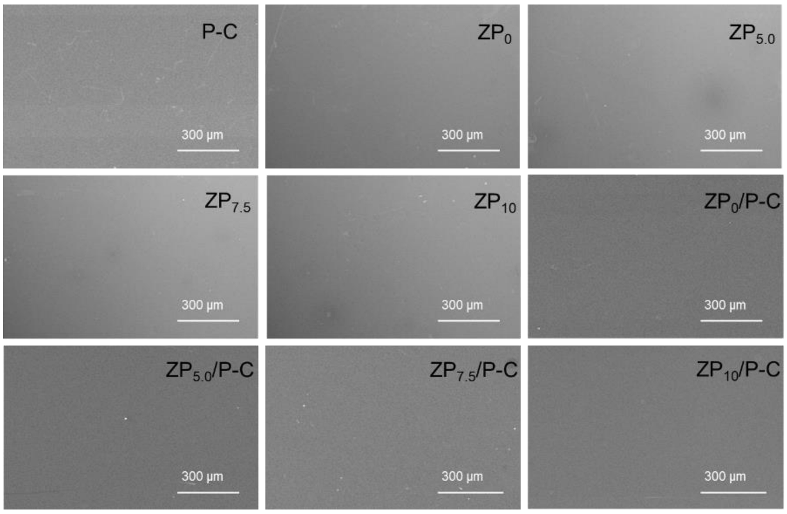

Figure S1. Surface SEM images of the different films

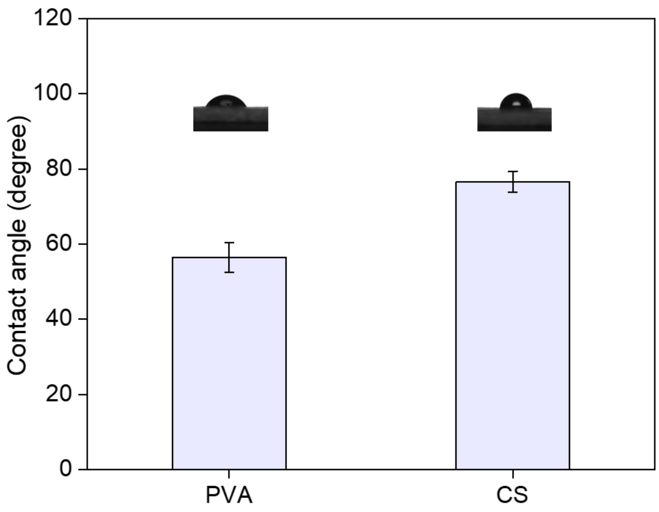

Figure S2. Contact angle of PVA and CS films

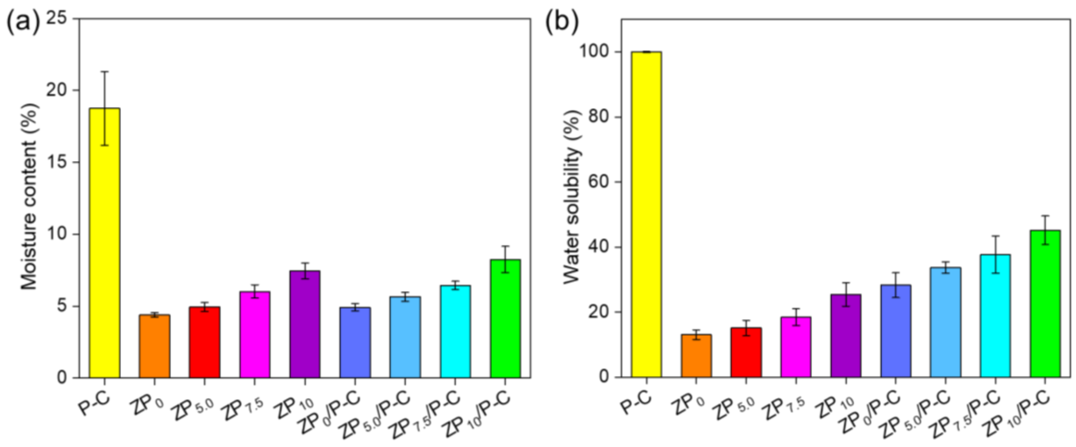

Figure S3. Moisture content and solubility of the different films.

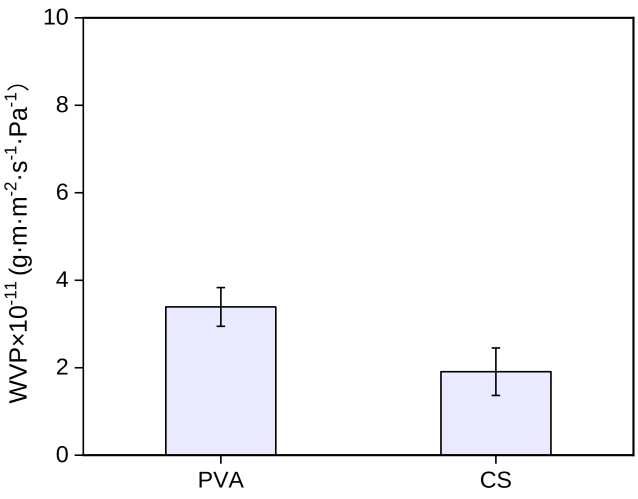

Figure S4. Water vapor permeability of PVA and CS films
